# Playing at the school table: Systematic literature review of board, tabletop, and other analog game-based learning approaches

**DOI:** 10.3389/fpsyg.2023.1160591

**Published:** 2023-06-02

**Authors:** Carla Sousa, Sara Rye, Micael Sousa, Pedro Juan Torres, Claudilene Perim, Shivani Atul Mansuklal, Firdaous Ennami

**Affiliations:** ^1^Lusófona University, CICANT, Lisbon, Portugal; ^2^LSBU, London Center for Business and Entrepreneurship Research, London, United Kingdom; ^3^Department of Peace Studies and International Development, University of Bradford, Bradford, United Kingdom; ^4^CITTA, Departamento de Engenharia Civil, Universidade de Coimbra, Coimbra, Portugal; ^5^HEI-Lab, Lusófona University, Lisbon, Portugal

**Keywords:** board games, tabletop games, analog games, game-based learning, education, systematic literature review

## Abstract

The unique characteristics of games have led scientific research to increasingly focus on their potential role in learning processes. Currently, their effectiveness in fostering experiential learning and skill acquisition in several areas is already supported by the existing evidence, mainly about the potential of digital games. Paradoxically, the current post-digital era seems to have led to a growing popularity of analog games. The present Systematic Literature Review aimed to map the existing literature on the potential of board, tabletop, or other analog games in learning processes. It intended to systematize the contemporary state of the art (2012–2022) around the pedagogical role of these games, their effectiveness, the promoted learning outcomes, the methodological aspects of the interventions, the used games—including mechanics and other characteristics—and the current discussions around inclusion and accessibility in analog game-based learning. Adopting the PRISMA methodology, we searched ACM Digital Library, EBSCO, ERIC, Scopus—Elsevier, and Web of Science databases, as well as other peer-reviewed “grey literature” sources. The search resulted in an initial sample of 2,741 articles that was then screened by inclusion and exclusion criteria previously defined according to the research objectives. We obtained a final sample of 45 articles. To formulate the mapping of existing research, these studies were analyzed using a combination of statistical, content, and critical analysis procedures. The obtained results support the role of board, tabletop, and other analog games in educational contexts—based on their educational potential—with a broad range of knowledge, cognitive, and psychological outcomes. The study also emphasized the relevance of these games in the promotion of soft skills and other aspects typically associated with meaningful learning, such as engagement, satisfaction, flexibility, and freedom of experimentation. However, important limitations were found in a fair amount of the pedagogical approaches studied, which can be mostly attributed to the low prevalence of modern board games that relate what is intended to be learned to aspects of game design and have little to no consideration of accessibility and inclusion aspects in these studies.

## 1. Introduction

Digitalization has globally dominated most of the northern countries/continents, and large efforts are being undertaken for the southern countries/continents to follow the same path (Reis et al., 2020). Arguably, digitalization is considered synonymous to modernization, development, and high standards for production, culture, and wellbeing (Kwilinski et al., 2020). Although digitalization is a global trend, there are indications that analog technologies are still popular despite being considered obsolete by modern technological standards. This raises the question of whether people are experiencing negative effects from over-digitalization and if we are living in a post-digital age (Cramer, 2015). The effects of reactions against digitalization were identified even before the COVID-19 pandemic, which eventually forced millions of people to rely on digital technologies. Aligned with this trend of reactions to over-digitization, board games, tabletop games, card games, and many other analog games are as popular as ever (Konieczny, 2019; Booth, 2021), especially due to new types of games like modern tabletop and board games (Woods, 2012; Rogerson and Gibbs, 2016; Arnaudo, 2018).

Playing and learning are almost interchangeable concepts and one of the most studied relationships since the early days of developmental psychology, by authors such as Jean Piaget or Lev Vygotsky. Playful activities have been studied as pillars for healthy minds in all ages, considering their ability to allow experimentation, often at a higher level of complexity than the “real world.” Thus, as scientific research has advanced, there has been an understanding of the potential of play to capitalize on brain plasticity to enhance human development (Hodent, 2021).

Games are one of the many playful activities humans can perform and, in this case, endowed with very specific characteristics. This includes interactivity, goal orientation (Costikyan, 2002), motivation through failure, or immediate feedback (Boyle et al., 2016). According to Errity et al. (2016), when a person plays a game, three types of consequences occur: (a) psychological gratifications; (b) altered states of consciousness—based on phenomena such as presence (Lombard and Ditton, 1997), immersion (Slater, 1999), and flow (Csikszentmihalyi, 1990); or (c) learning processes and enhanced adaptive skills.

Game-based learning (GBL) can be defined as using games to facilitate a learning experience. GBL takes the social experience of playing a game to a learning environment, allowing educators to use game mechanics for promoting specific activities to attain defined learning outcomes (Plass et al., 2015). Research has been able to support the potential of digital games to foster consistent learning gains in a broad range of areas of implementation, and as transversal approaches, effective in educational settings (Sousa and Costa, 2018).

So, we can say that the state of the art is already cohesive enough to support the potential of games in learning processes (Arnab et al., 2014; Abdul Jabbar and Felicia, 2015; Qian and Clark, 2016), although the supremacy of digital games is also an aspect to be considered (Naik, 2014) and tackled. The present Systematic Literature Review (SLR) aims to map the existing literature on the potential of board, tabletop, or other analog games in learning processes through the operationalization of different specific objectives.

– Research Objective 1 (RO1): To explore the effectiveness of analog GBL.
– RO2: To analyze the adopted research designs and other methodological aspects of the existing approaches to analog GBL.
– RO3: To explore the main outcomes of analog GBL, including learning outcomes, psychological, and cognitive outcomes.
– RO4: To explore the used games and mechanics.
– RO5: To explore how research in the field of analog GBL has been operationalizing inclusion and accessibility measures.

## 2. Methods

### 2.1. Eligibility criteria

The search strategy of the present SLR was developed considering the PRISMA 2020 statement guidelines for the reporting of systematic reviews (Page et al., 2021). Considering the research objectives described above, inclusion and exclusion criteria were formulated to support the selection process of the scientific articles. These criteria also considered the increased quality of systematic reviews that are based only on the most recent evidence (Schlosser, 2007).

The present SLR includes peer-reviewed empirical research published between 2012 and 2022 that approaches the potential of analog games for learning purposes. It is important to clarify that “analog games” are used in this SLR as a broad notion that can contain categories such as “board games,” “tabletop games,” “card games,” “dice games,” or any other that does not imply the usage of digital technologies. Consequently, all secondary studies—e.g. other literature reviews or meta-analyses—were excluded from the sample, as well as theoretical or position papers. Studies approaching the learning potential of digital, or hybrid games were also excluded.

### 2.2. Information sources

The systematic search was conducted in the scientific databases defined by the research team. This included ACM Digital Library, EBSCO, ERIC, Scopus—Elsevier, and Web of Science. Considering the nature of the study, and the potential of evidence emerging from other sources besides the exclusively academic ones, ResearchGate was also included as an information source and data were also requested from networks of academics in the field of GBL. This intended to broaden the scope of the review while providing a more comprehensive notion of the available evidence (Mahood et al., 2013).

### 2.3. Search strategy

In terms of the search strategy, the search equation was composed as follows: (analog OR analogue OR board OR card OR dice OR tabletop) AND (game OR gaming OR games) AND (learning OR education). Subsequently, some filters were applied, according to the possibilities offered by each database, namely: “peer-reviewed research only;” “English only,” or “search in abstract and title.” The time interval for the publications was also applied, in this case between 2012 and 2022. The systematic searches were conducted on September 11, 2022.

### 2.4. Selection process

**Figure 1:**
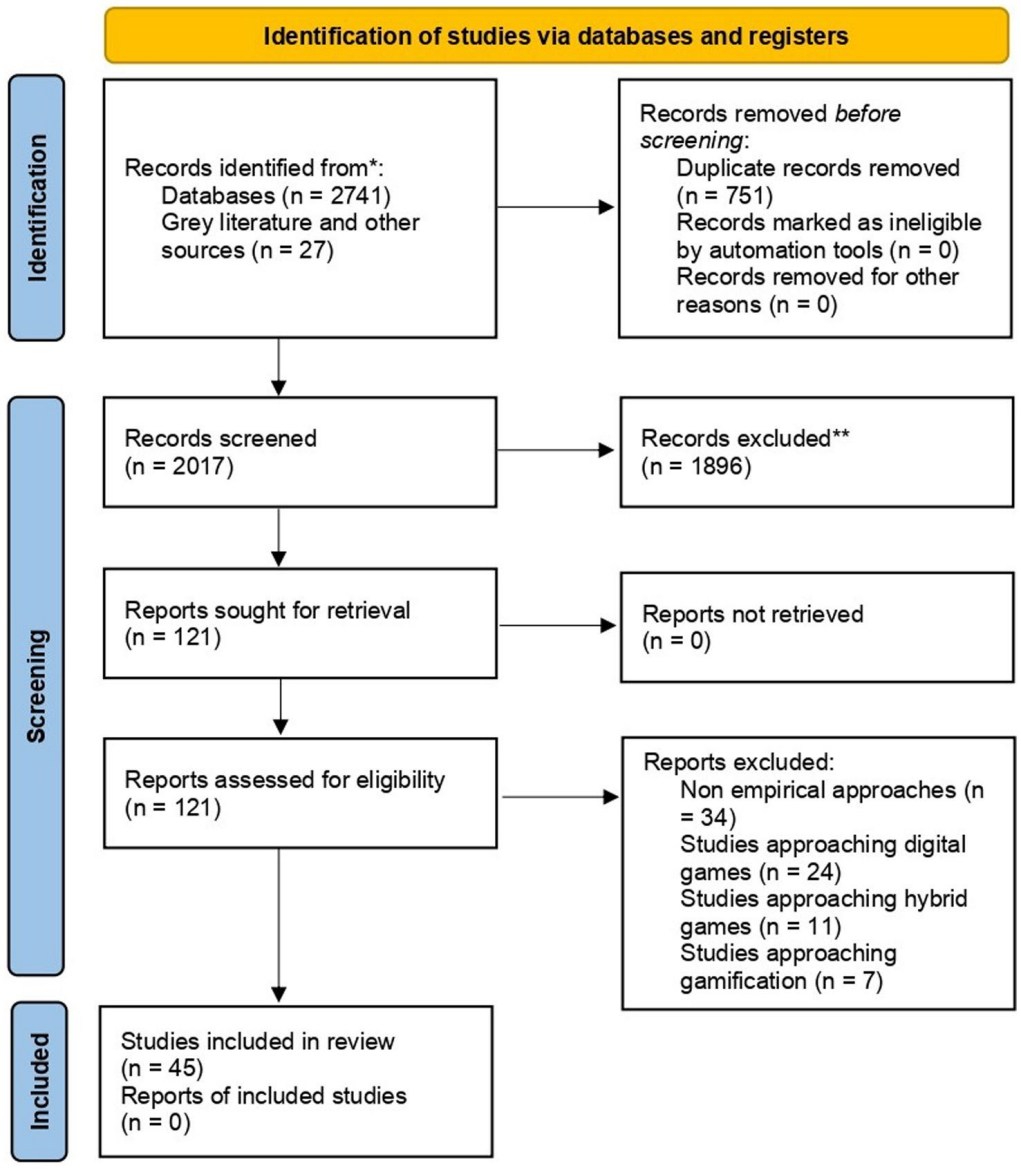
PRISMA (Page et al., 2021) flowchart of the selection process.

The selection process throughout the final sample is represented in the flowchart in Figure 1.

The identification phase was developed by applying the search strategy to the information sources and retrieving the obtained data. The screening phase was developed by applying the inclusion and exclusion criteria to the initial sample of studies (*N* = 2017), only by reading title and abstract. Peer-reviewed research developed between 2012 and 2022, adopting an empirical research design to the study of analog games for learning purposes was included in the sample. On the opposite side, other secondary research (literature reviews, meta-analysis, or others), theoretical studies, and the ones based in the usage of digital or hybrid games were excluded from the sample. The eligibility phase was developed by applying the same procedure but by thoroughly analyzing the full paper of each study in that stage of the sample (*N* = 121). Through this procedure, the final sample of 45 studies was reached, as represented in Table 1. All the described phases were conducted by two researchers, as a strategy to tackle bias in sample inclusion, with the support of Rayyan,^1^ and disagreements were solved through conceptual discussion meetings.

**Table 1:** Final sample of studies, year, author(s), and titles (*N* = 45).

Study	Year	Author(s)	Title
1	2012	Wangenheim et al.	DELIVER!—An educational game for teaching earned value management in computing courses
2	2013	Denning et al.	Control-Alt-Hack: the design and evaluation of a card game for computer security awareness and education
3	2013	Liu and Chen	The effect of game-based learning on students’ learning performance in science learning – a case of "Conveyance Go"
4	2013	Paris and Yussof	Use of “Time Trap Board Game” to teach grammar
5	2015	Kobzeva	Scrabble as a tool for engineering students’ critical thinking skills development
6	2016	Gilliam et al.	LifeChanger: a pilot study of a game-based curriculum for sexuality education
7	2016	Sardone and Devlin-Scherer	Let the (Board) games begin: creative ways to enhance teaching and learning
8	2017	Carreira et al.	"The Celsius Game: an experiential activity on management education simulating the complex challenges for the two-degree
9	2017	Chappin et al.	Teaching sustainability to a broad audience through an entertainment game e The effect of Catan: oil springs
10	2018	Azizan et al.	"Improving teamwork skills and enhancing deep learning via development of board game using cooperative learning method in Reaction Engineering course"
11	2018	Despeisse	"Teaching sustainability leadership in manufacturing: a reflection on the educational benefits of the board game Factory Heroes"
12	2019	Giles et al.	Creating a library orientation card game to reach new transfer students
13	2019	Lavender et al.	Evaluation of an educational board game to improve use of the partograph in sub-Saharan Africa: a quasi-experimental study
14	2019	Luchi et al.	Increased learning by using board game on muscular system physiology compared with guided study
15	2019	Sarinho	Masters of the process: a board game proposal for teaching software management and software development process
16	2020	Armstrong	Playing settlers of catan enhances student learning of probability in liberal arts mathematics
17	2020	Casey et al.	Maternal use of math facts to support girls' math during card play
18	2020	Hart et al.	Riskio: a serious game for cyber security awareness and education
19	2020	Martindale and Weiss	“Taphonomy: Dead and fossilized”: a new board game designed to teach college undergraduate students about the process of fossilization
20	2020	Severengiz et al.	Serious game on factory planning for higher education
21	2021	Bernardo and González	Chemical battleship: discovering and learning the periodic table playing a didactic and strategic board game
22	2021	Ezezika et al.	The pedagogical impact of board games in public health biology education: the Bioracer Board Game
23	2021	Ghiga et al.	PIPDeploy: development and implementation of a gamified table top simulation exercise to strengthen national pandemic vaccine preparedness and readiness
24	2021	Hsu et al.	Behavioral-pattern exploration and development of an instructional tool for young children to learn AI
25	2021	Kurisu et al.	Development of board game to encourage life cycle thinking, and trial with university students in Japan
26	2021	Lew and Saville	Game-based learning: teaching principles of economics and investment finance through Monopoly
27	2021	Mildenhall et al.	The honey bees game: engaging and inspiring the community with STEM
28	2021	Minato et al.	Developing a remote team training program based on the space flight resource management model
29	2021	Parrondo et al.	Sustainable sea: a board game for engaging students in sustainable fisheries management
30	2021	Rahimi and Kim	Learning through redesigning a game in the STEM Classroom
31	2021	Vasconcelos and Seingyai	Planning for sustainable development: a simulation game
32	2021	Vázquez-Vílchez et al.	Using a cooperative educational game to promote pro-environmental engagement in future teachers
33	2022	Bressler et al.	“What if We Explore...” promoting engaged learning and collaboration with mountain rescue
34	2022	Chang Y. S et al.	Effects of board game play on nursing students’ medication knowledge: a randomized controlled trial
35	2022	Chang C. H. S. et al.	"Design and evaluation of a multi-sensory scaffolding gamification science course with mobile technology for learners with
36	2022	Mavroudi et al.	A card game for designing activities for technology-enhanced learning in higher education
37	2022	Niedderer et al.	This is me: evaluation of a boardgame to promote social engagement, wellbeing and agency in people with dementia through mindful life-storytelling
38	2021	Veldthuis et al.	A quest to engage computer science students: using dungeons & dragons for developing soft skills
39	2020a	Sousa	Modern serious board games: modding games to teach and train civil engineering students
40	2020b	Sousa	A planning game over a map: playing cards and moving bits to collaboratively plan a city
41	2020c	Sousa	Fast brainstorm techniques with modern board game adaptations for daily uses in business and project managing
42	2021a	Rosa et al.	Critical thinking, empathy and problem solving using a modern board game
43	2021b	Rosa et al.	Empathy, creativity, and feelings using a modern board game
44	2022	Sousa et al.	Fast serious analog games in planning: the role of non-player participants
45	2021	Vasconcelos	Collaborating: modern board games and collaboratories as a tool for capacity building

### 2.5. Analysis and synthesis of results

To analyze the obtained sample of studies (*N* = 45), their information was coded, considering the most relevant categories to the research aims defined above. This included: subject area of the publication; sample size and characteristics; main goal; aimed learning, cognitive, and psychological outcomes; used games and mechanics; research design; assessment procedures; inclusion and accessibility features; and effectiveness in the learning process. Board Game Geek (BGG) database and Scimago Journal & Country Rank were adopted as additional sources to support the coding of game mechanics and subject area of the publication, respectively. After coding the 45 papers for each specific category, data were analyzed through descriptive statistical analysis procedures, supported by Statistical Package for the Social Sciences (SPSS), version 26. Risk of bias was addressed through a two-coder system, with a junior researcher and senior research coding similar materials.

Due to their nature, the results categories “Impact and Effectiveness” and “Inclusion and Accessibility” were summarized through critical analysis.

## 3. Results

### 3.1. Publication characteristics

**Figure 2:**
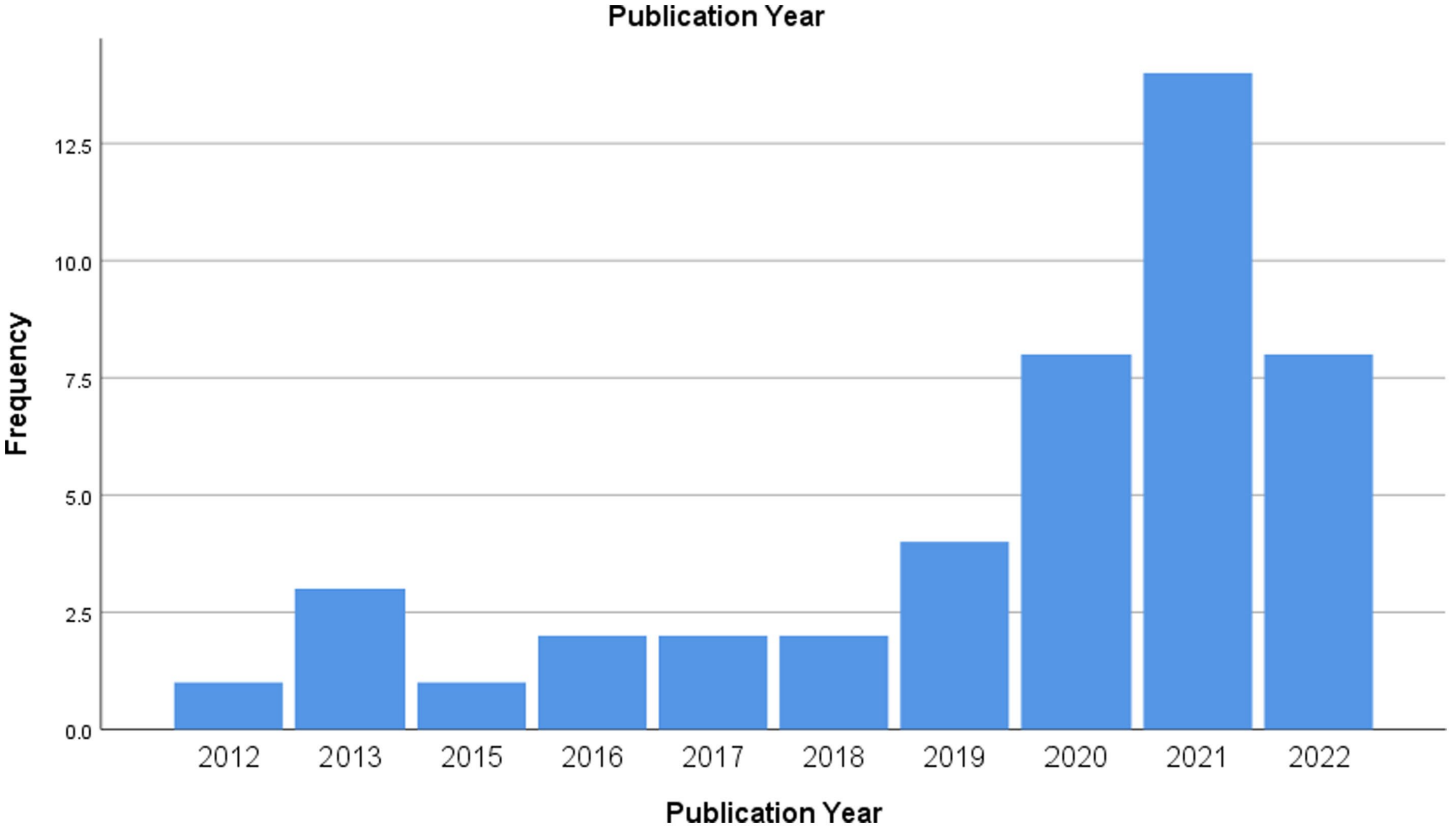
Publication year of the studies in the sample (*N* = 45).

To draw a general panorama of research in the area in terms of its chronological dimension and scientific domains, the years and areas of publication of the respective journals were analyzed, as shown in Figure 2.

These data show that 2021 was the year with a higher number of published studies in the sample (*N* = 14; 31.10%), followed by 2020 and 2022, with eight studies (17.80%) each. The years of 2012 and 2015 were the years with less scientific production in the sample (*N* = 1; 2.20%), apart from 2014, which, of this range, was the year that did not record any publication in the sample. It is important to highlight that the systematic database search was performed in September 2022, which may leave out some publications made in this interval.

Regarding the scientific domains of each publication, two levels of analysis were adopted. First, each article was classified according to the main scientific area of the journal in which it was published. Second, the articles whose journals had a quartile (*N* = 34) were classified considering the category/sub-area of its highest quartile, enabling a greater level of detail in the analysis. Both procedures were conducted in December 2022, according to the Scimago Journal & Country Rank data.

In the first level of analysis, a total of 10 scientific areas were coded, with the following results: social sciences (*N* = 23; 51.10%); computer sciences (*N* = 8; 17.80%); business, management and accounting (*N* = 3; 6.70); engineering (*N* = 3; 6.70); environmental science (*N* = 2; 4.40%); nursing (*N* = 2; 4.40%); biochemistry, genetics, and molecular biology (*N* = 1; 2.20%); chemical engineering (*N* = 1; 2.20%); medicine (*N* = 1; 2.20%); and Psychology (*N* = 1; 2.20%). In the second level of analysis, a total of 17 categories or sub-areas were coded, according to the quartiles, resulting in the data presented in Table 2.

**Table 2:** Categories/sub-areas of the journals, according to higher quartile (*N* = 34).

Category/Sub-area of the higher quartile	*N*	%
Education	10	29.40
Social science (miscellaneous)	7	20.60
Communication	2	5.90
Computer science (miscellaneous)	2	5.90
Computer science applications	1	2.90
Computer networks and communications	1	2.90
Pediatrics, perinatology, and child health	1	2.90
Renewable energy, sustainability, and the environment	1	2.90
Chemical engineering (miscellaneous)	1	2.90
Library and information sciences	1	2.90
Maternity and midwifery	1	2.90
Developmental and educational psychology	1	2.90
Artificial intelligence	1	2.90
Molecular medicine	1	2.90
Safety, risk, reliability, and quality	1	2.90
Human-computer interaction	1	2.90
Issues, ethics, and legal aspects	1	2.90
Total	34	100.00

It is possible to highlight education in these sub-areas (*N* = 10; 29.40%), followed by the general or multidisciplinary areas of the social sciences (*N* = 7; 20.60). In the remaining data, it is possible to verify quite dispersion, highlighting that only communication and general fields of computer science present more than one study (5.90%).

### 3.2. Study participants

The present SLR had a total sample size of 3,550 subjects, with each article’s sample ranging from six to 760 participants (*M* = 80.68; *SD* = 143.88). Subsequently, studies were categorized considering their sample of participants, to understand the most widely covered players in the analog game-based learning field, as shown in Table 3.

**Table 3:** Approached sample of each study (*N* = 45).

Approached sample	*N*	%
Higher education students	24	53.30
Elementary education students	5	11.10
Secondary school students	4	8.90
General population (anyone or not specified)	3	6.70
Teachers and/or lecturers	2	4.40
Mixed (higher education students and lecturers)	2	4.40
Mixed (higher education students and qualified professionals)	2	4.40
Mixed (higher education and secondary students)	1	2.20
People with dementia	1	2.20
People working in business	1	2.20
Total	45	100.0

When observing the data, it is possible to highlight that most studies adopt as sample higher education students (*N* = 24; 53.30%). This result becomes even more significant if we consider samples that combine students in higher education with other population groups—lecturers, qualified professionals, and secondary students—in which case it becomes 64.44% (*N* = 29) of all studies.

### 3.3. Games and learning

According to the content analysis methodology defined above, three possible types of outcomes of the game-based approaches described in the articles were specified: (a) learning; (b) cognitive; and (c) psychological. While recognizing the clear intersection between these outcomes, this methodological decision was intended to ensure a better understanding of the practical possibilities of these pedagogical strategies.

For each article, the main learning outcome was coded, considering the information provided by the authors. Then, up to two additional outcomes were coded if they were explicitly mentioned or described in the article. The same process was carried out for cognitive and psychological outcomes. The result of the total number of coded learning outcomes is shown in Table 4.

**Table 4:** Total coded learning outcomes (*N* = 62).

Learning outcome	*N*	%
Collaboration	10	16.14
Communication	9	14.52
Science	8	12.90
Sustainability	8	12.90
Computer science	4	6.45
Engineering	3	4.84
Language	3	4.84
Mathematics	2	3.23
STEM	2	3.23
Planning	2	3.23
Sexuality education	1	1.61
Management	1	1.62
Digital literacy	1	1.61
Paleontology	1	1.61
Organizational skills	1	1.61
Finance	1	1.61
21st century skills	1	1.61
Critical thinking	1	1.61
Medicine	1	1.61
Soft skills (in general)	1	1.61
Storytelling	1	1.61
Total	62	100

As in the data for the scientific areas, also for the learning outcomes, we can find significant dispersion, with board games being used to promote a range of different learning experiences. Nevertheless, it is possible to highlight the relevance of the so-called soft skills, particularly communication (*N* = 10; 16.14%) and collaboration (*N* = 9; 14.52%), with one paper (1.61%) also mentioning the promotion of soft skills in general. The interventions targeted to science learning (*N* = 8; 12.90%) and to the promotion of sustainability-driven attitudes (*N* = 8; 12.90%) were also expressive in the total of coded learning outcomes.

Two different types of cognitive outcomes were coded, with a total of four mentions in the sample of articles. The most prevalent was memory (*N* = 3; 75.00%), followed by problem-solving (*N* = 1; 25.00%). Psychological outcomes were mentioned in the sample in eight different occasions, and it was possible to obtain the following results in this field: creativity (*N* = 4; 50.00%); empathy (*N* = 2; 25.00%) self-confidence (*N* = 1; 12.50%); and wellbeing (*N* = 1; 12.50%).

Regarding the games adopted in the studies, it is possible to mention that most of them used games that were specifically created for research purposes (*N* = 32; 71.10%), while the remaining 13 (28.90%) used commercial games, which can easily be purchased in shops. The used commercial games included: Telestrations (*N* = 3); Catan (*N* = 2); Dixit (*N* = 2); Codenames (*N* = 1); Control-Alt-Hack (*N* = 1); Dungeons & Dragons (*N* = 1); Just One (*N* = 1); Magic Maze (*N* = 1); Monopoly (*N* = 1); Scrabble (*N* = 1); Spyfall (*N* = 1); Steam (*N* = 1); and Town Center (*N* = 1).

For each game, the main game mechanic was coded, according to the author’s descriptions and the Board Game Geek (BGG) database of mechanisms. Then, up to two additional mechanics were coded per game. In the studies that used more than one game, the most mentioned was considered as the main one. However, in the total number of mechanics, all games were considered. Table 5 illustrates the 38 coded mechanics, with a total of 101 mentions.

**Table 5:** Total coded game mechanics (*N* = 101).

Game mechanic	*N*	%
Dice-rolling	13	12.87
Events	11	10.89
Cooperative game	8	7.92
Team-based game	8	7.92
Roll/Spin and move	6	5.94
Hand management	5	4.95
Role-playing	4	3.96
Grid movement	3	2.97
Income	3	2.97
Simulation	3	2.97
Communication limits	3	2.97
Drawing	3	2.97
Square grid	2	1.98
Auction/Bidding	2	1.98
Memory	2	1.98
Hexagon grid	2	1.98
Questions and answers	2	1.98
Pick-up and deliver	1	0.99
End game bonuses	1	0.99
Semi-cooperative game	1	0.99
Action points	1	0.99
Player judge	1	0.99
Simultaneous action selection	1	0.99
Deduction	1	0.99
Secret unit deployment	1	0.99
Pattern building	1	0.99
Tile placement	1	0.99
Acting	1	0.99
Area majority/Influence	1	0.99
Storytelling	1	0.99
Elapsed real time ending	1	0.99
Paper-and-pencil	1	0.99
Action selection restriction	1	0.99
Exchanging	1	0.99
Negotiation	1	0.99
Card play conflict resolution	1	0.99
Pattern recognition	1	0.99
Variable player powers	1	0.99
Total	101	100

Dice-rolling was the most common mechanic in the used games (*N* = 13; 12.87), followed by events (*N* = 11; 10.89), i.e., actions that happen outside of the player’s control causing immediate effect on the gameplay. Cooperative game and team-based game were also prevalent mechanics, with eight games each (7.92%).

Thereafter, cross-tabulation was used to understand the cross-prevalence between the main mechanics of each game and the study’s main learning outcome. Most results were equal to zero or one, except for:

– Three studies aimed at the promotion of sustainability used one or more games with dice-rolling as a mechanic;
– Two studies in the field of computer sciences used one or more games with events as a mechanic;
– Two studies aimed at the promotion of scientific knowledge used one or more games with roll/spin and move as a mechanic; and
– Two studies aimed at the promotion of sustainability were cooperative games.

A similar procedure was developed for cognitive and psychological outcomes. The specific game mechanics involved in the promotion of these variables are expressed in Table 6.

**Table 6:** Cross-tabulation of cognitive and psychological outcomes with game mechanics.

	Memory	Problem-solving	Self-confidence	Creativity	Empathy	Wellbeing	Total
Dice-rolling	2	0	0	0	0	1	3
Grid movement	0	0	1	0	0	0	1
Hand management	1	0	0	0	0	0	1
Communication limits	0	1	0	0	1	0	2
Role-playing	0	0	0	1	0	0	1
Action points	0	0	0	1	0	0	1
Storytelling	0	0	0	1	0	0	1
Drawing	0	0	0	1	0	0	1
Total	3	1	1	4	1	1	11

### 3.4. Adopted research approaches

From the analysis of the methodological approach of each study, it is possible to highlight a predominance of quantitative studies (*N* = 24; 53.30%) in the field of board games and learning. Nevertheless, it is also possible to highlight a large number of mixed methods studies (*N* = 18; 40.00%), in which quantitative and qualitative approaches were integrated. The exclusive use of qualitative methods appeared as less expressive in the sample (*N* = 3; 6.70%).

With regard to the type of evaluation adopted in each research design, namely the moment or moments in which it was implemented, the results are shown in Table 7.

**Table 7:** Assessment models implemented in each study (*N* = 45).

Assessment implemented in study	*N*	%
Post intervention	19	42.20
Pre and post intervention	7	15.60
Pre and post intervention with performance assessment	6	13.30
Performance (during intervention)	5	11.10
Performance and post intervention	4	8.90
Pre and post with control group (experimental)	4	8.90
Total	45	100.00

From these results, it is possible to highlight that most studies (*N* = 19; 42.20%) assessed learning through a post intervention approach, i.e., after playing the game. Studies applying pre and post intervention assessments—i.e., before and after playing the game or games—were also very prevalent. This was done either exclusively (*N* = 7; 15.60%) or integrated with in-game performance assessment (*N* = 6; 13.60%). Moreover, there were also four studies (8.90%) where pre and post assessment was conducted in the context of experimental randomized controlled trials.

### 3.5. Impact and effectiveness

Most studies in the sample reported analog GBL as an effective pedagogical tool with an impact on the learning, cognitive, and psychological levels. These include the learning outcomes systematized in Table 4, cognitive outcomes—such as memory and problem-solving—and psychological outcomes, such as creativity, empathy, self-confidence, and wellbeing.

The studies included in the sample also addressed how board games can promote changes in learning processes in other aspects, including how these media tends to promote increased learners’ engagement (Sousa, 2020a,c; Ezezika et al., 2021; Ghiga et al., 2021; Bressler et al., 2022; Sousa et al., 2022), satisfaction (Sarinho, 2019; Sousa, 2020a), and overall facilitating the learning process (Sarinho, 2019; Bernardo and González, 2021). According to Giles et al. (2019, p. 9), board games tend to create learning opportunities that are described as “fun, social, flexible, and inexpensive.” This notion might also explain their role in the elimination of barriers identified in the learning process (Despeisse, 2018), as well as in fostering not only knowledge acquisition, but also behavioral change (Chappin et al., 2017).

Furthermore, the possibility to include learners in the building of their own knowledge is also pointed as a pillar of analog GBL by the different studies (Gilliam et al., 2016; Sousa, 2020b,c; Vasconcelos and Seingyai, 2021), which will address such crucial aspects of this premise as freedom of experimentation (Rosa et al., 2021b). More positive attitudes toward the learning process as a whole also seem to result from the use of board games in the educational context (Liu and Chen, 2013; Sardone and Devlin-Scherer, 2016).

Authors like Bartolucci et al. (2019) questioned the possibility of people becoming smarter by playing these games. This is something we should approach carefully considering the effect of several impeding variables, although the results of analog game usage point to this. To underpin evidence-based interventions in this field, a meta-analysis of the synthetized data is relevant. However, only four studies (Luchi et al., 2019; Armstrong, 2020; Ezezika et al., 2021; Chang Y. S. et al., 2022) had adequate methodological characteristics and given their disparities in terms of research design, it was not possible.

### 3.6. Accessibility and inclusion

According to Booth (2021, p. 189), the board game communities tend to be characterized by their “overall friendliness and welcoming nature,” aligned with an industry that is mostly willing to receive players’ feedback and hear their needs. In the present SLR, it seemed relevant to study how research in the area has followed this premise, by operationalizing principles of inclusion and accessibility. So, even though analog GBL itself may be linked to a view of simplifying learning and therefore promoting inclusion, we checked how often and how these aspects were mentioned in the studies.

A total of six studies (13.33%) specifically mentioned accessibility or inclusion concerns, approaching either literacy issues (Denning et al., 2013; Hart et al., 2020), specific audiences (Chang C. H. S. et al., 2022; Niedderer et al., 2022), or inclusive learning in general (Sousa, 2020c; Veldthuis et al., 2021).

Both Denning et al. (2013) and Hart et al. (2020) applied computer security awareness board games, promoting inclusion through a continuous effort to make them accessible to individuals with low digital literacy. Chang C. H. S. et al. (2022) made their study with blind learners as a main audience, while Niedderer et al. (2022) did the same but with older adults with dementia. In the second study, inclusive principles were also considered a pillar for the game design, since these individuals were considered as co-designers, and dementia was a creative trigger instead of a barrier (Niedderer et al., 2022). Considering the results of Sousa (2020c) and Veldthuis et al. (2021), board games can foster a sense of inclusion in the learning process in general, either because they promote a broad set of soft skills, or because they can support people who do not necessarily have a specific disability or condition—such as someone who is shy or a divergent thinker.

## 4. Discussion

The present study aimed to systematize the existing literature on the potential of board or other analog games in learning processes, with the overall results pointing toward the evidence of their relevant role in educational processes. Although the first objective of this research (RO1) was to study the effectiveness of games in processes, the results obtained seem to transcend the quantitative-qualitative debate in this sense. Beyond the quantitative aspects of the knowledge that was acquired, this research corroborates the role of board games in promoting aspects typically associated with meaningful learning, such as engagement, satisfaction, flexibility, or freedom of experimentation.

In a more detailed manner, and regarding the publication year and research landscape of analog GBL, it should be noted that it seems to accompany the previously approached growing popularity of modern tabletop and board games (Woods, 2012; Rogerson and Gibbs, 2016; Arnaudo, 2018). In the scientific domain, the obtained results seem to align with the diversity previously described for game studies or ludology in general. It is essential to underline that subjects like social sciences/education, computer sciences or a specific field of knowledge might be prioritized depending on the study and the way the game was framed. Thus, this relationship seems to be ideologically framed, depending on how one analyzes the relationship between game and play (Frasca, 2007).

At a methodological level—as and proposed by RO2—the sample of articles collected presents a gap that should be highlighted. Most participants included in the different studies are higher education students, which, although comprehensible for feasibility, raises two types of issues: (a) a lack of representation of voices in research on board games and learning; and (b) some homogeneity in the complexity of the proposed in-game pedagogical objectives. The approaches are characterized by their diversity—although there is a predominance of quantitative approaches, there is a high frequency of mixed approaches. Regarding research design, two main aspects were noted: (a) the existing difficulty in studying the effectiveness of board GBL approaches given the low prevalence of experimental studies with standards that allow the conduction of a meta-analysis and (b) the expressiveness of post assessment in the studies, which is in line with the importance of debriefing in GBL.

The present study also corroborates the potential of analog game-based approaches in learning a multitude of specific content or skills. This aligns with findings from previous studies on the potential of digital games (Sousa and Costa, 2018). However, in the case of analog games, their potential in promoting soft skills, with a particular focus on communication and collaboration, seems to stand out as the main outcomes, as proposed in RO3. It is relevant to emphasize its potential in stimulating psychological and cognitive variables that underlie teaching and learning processes, including creativity, memory, empathy, problem-solving, self-confidence, and wellbeing.

Regarding the fourth research objective (RO4) and the study of the used games, this sample showed that most of them were created for the project at stake. This dominance might be problematic and says little about the potential of these games, considering that their design dimensions are unknown. Using dice-rolling mechanics is not enough to classify the type of game involved. Even when considering the BGG databases, once again the most common game mechanic/mechanism is dice-rolling (Samarasinghe et al., 2021). This feature includes many older games, like the classic role and moves games that have been unaltered since the XIX century from a game design perspective (Woods, 2012). So, the games from the sample might not deliver the same experiences as the most modern analog ones.

It was notorious that the game approaches from the sample were complemented with other auxiliary activities. This was expected because we are dealing with games developed and played to deliver more than entertainment, cases of GBL, and overall serious games. The most well-known literature in the field of serious games argues that these games have a higher impact when combined with other activities (Wouters et al., 2013) and that they demand facilitation and debriefing to assure that the serious of the objectives of the game are met (Crookall, 2010).

This aspect seems to extend to a certain arbitrariness between game mechanics and learning. In other words, the results of this study emphasize a lack of congruence between game systems and what is intended to be taught, with these contents being much more associated with the game theme than with its mechanisms and dynamics. In this sense, more studies are needed to establish clear parallels between game mechanics and the aspects of learning they are intended to promote. The study developed by Vita-Barrull et al. (2022)—in which some board game mechanics and cognitive processes were mapped—is an example of the kind of results that are intended to be achieved, also in the educational field.

Considering the fifth research objective (RO5), it was possible to detect a small number of mentions of accessibility and inclusion aspects in the studied literature. Although the board game community and industry are seen as particularly inclusive (Booth, 2021), inclusion and accessibility appear to be a minor concern of analog GBL research. Nevertheless, from the results obtained, the potential in promoting a sense of inclusion in the learning process, which can be provided by board games, is also highlighted.

## 5. Conclusion

The results obtained in this study support the role of analog games in educational processes, highlighting this area as increasingly popular in scientific research and widely multidisciplinary. This study also systematized evidence on the potential of these games in promoting different skills and knowledge, with a particular focus on soft skills. In a broader sense, board games seem to have a relevant role in the promotion of several aspects that are transversal to the success of the learning process, both at psychological and cognitive levels.

The sample of articles analyzed allowed us to verify the existence of some particularities and limitations in this area of research. These limitations include heterogeneity of research designs, which hinders the statistical summarization of effectiveness data, still relevant in the context of policymaking. In addition, the analog GBL approaches seem to use mainly games produced in research contexts, making it difficult to analyze their game design and hampering their wide dissemination among educational stakeholders. There appear to be limited connections between the learning contents and specific aspects of gameplay such as game mechanics, thus restricting the potential of game design in learning.

Moreover, the statistics that were calculated were based on a small sample of studies, which made it difficult to identify clear trends. Therefore, it would be beneficial to establish connections that could create more coherent frameworks for analog game-based learning. These frameworks would link populations, learning outcomes, games, and their characteristics.

Future studies should include non-academic approaches to analog GBL and its potential social impact, ensuring a broader coverage of the state-of-the-art that bridges the gap between academia and civil society in this area. It will also be crucial to reflect on the potential of games for inclusion and exclusion from the learning process, depending on the degree of representation, diversity, and accessibility that is implemented in each approach.

## Author contributions

CS, SR, and MS: conceptualization. CS and SM: data curation and software. PT and CS: formal analysis. SR and CS: funding acquisition, project administration, methodology, and supervision. CS, SR, MS, and PT: investigation. CS, PT, CP, and FE: resources. CS and MS: validation and visualization. CS, MS, CP, SR, and FE: writing–original draft. CS, SR, MS, PT, CP, SM, and FE: writing–review and editing. All authors contributed to the article and approved the submitted version.

## Funding

The present work was developed in the scope of the Project Training the Educators to facilitate the Teaching and Assessment of Abstract Syllabus by the Use of Serious Games—TEGA (2020–1-UK01-KA203-079248), funded by the European Commission on the scope of Erasmus+ Program. The research team also acknowledges the funding by Fundação para a Ciência e para a Tecnologia (FCT) provided to CICANT R&D Unit (UIDB/05260/2020), on the scope of Verão com Ciência initiative, which allowed the inclusion of a research initiation grant holder as co-author of the present work.

## Conflict of interest

The authors declare that the research was conducted in the absence of any commercial or financial relationships that could be construed as a potential conflict of interest.

## Publisher’s note

All claims expressed in this article are solely those of the authors and do not necessarily represent those of their affiliated organizations, or those of the publisher, the editors and the reviewers. Any product that may be evaluated in this article, or claim that may be made by its manufacturer, is not guaranteed or endorsed by the publisher.

## Footnotes

^1^ https://www.rayyan.ai/
